# Novel AF1q/MLLT11 favorably affects imatinib resistance and cell survival in chronic myeloid leukemia

**DOI:** 10.1038/s41419-018-0900-7

**Published:** 2018-08-28

**Authors:** Wei Li, Min Ji, Fei Lu, Yihua Pang, Xin Dong, Jingru Zhang, Peng Li, Jingjing Ye, Shaolei Zang, Daoxin Ma, Chunyan Ji

**Affiliations:** Department of Hematology, Qilu Hospital, Shandong University, Jinan, 250012 China

## Abstract

Tyrosine kinase inhibitor treatment of chronic myeloid leukemia (CML) has demonstrated beneficial effects. However, resistance to tyrosine kinase inhibitors and disease relapse are still a challenge for CML therapy. In this study, we analyzed bone marrow samples from 149 CML patients and 15 control donors, and investigated the affect of AF1q on CML cell survival and engraftment in vitro and in vivo. We found that *AF1q/MLLT11* expression was significantly upregulated in CML patients, especially in CD34^+^ CML cells. Elevated AF1q expression was associated with disease progression. Knockdown of AF1q enhanced imatinib sensitivity, induced apoptosis, and suppressed growth in CML cells. Moreover, AF1q deficiency sensitized CD34^+^ CML cells to imatinib. In contrast, upregulation of AF1q promoted cell survival, protected CML cells from imatinib-induced apoptosis, and increased engraftment of CML cells in vivo. We further identified a positive correlation between *AF1q* and *CD44* expression in chronic phase CML patients and CD34^+^ CML cells. Importantly, AF1q contributes to imatinib-resistance in CML by regulating the expression of CD44. These findings reveal a novel BCR-ABL-independent pathway, AF1q/CD44, involves imatinib resistance in CML, thus representing a potential therapeutic target for imatinib-resistant CML patients.

## Introduction

Chronic myeloid leukemia (CML) is a clonal hematopoietic stem cell (HSC) disorder characterized by the t(9;22)(q34;q11) translocation, which results in formation of the fusion oncogene *BCR-ABL*^1,2^. The BCR-ABL protein has constitutive tyrosine kinase activity that directs HSC differentiation toward myeloid progenitors and differentiated myeloid cells expansion, and is essential for the growth of CML cells^3,4^. The BCR-ABL specific tyrosine kinase inhibitors (TKIs), such as imatinib (IM), are highly effective in the treatment of CML, leading to complete cytogenetic responses (CCyR) in a majority of chronic phase (CP) CML patients^5^. However, these TKI therapies are not curative. A subset of patients show variable degrees of resistance to currently available TKIs^6^, and even in patients with deep molecular responses, relapse occurs quickly in a majority of these patients after cessation of TKI therapy^7–9^. Furthermore, patients with advanced stages of CML are less responsive to TKI therapy and frequently develop TKI resistance^10,11^. The sources of disease persistence and relapse may include individual intolerance to TKIs, BCR-ABL mutation-related drug resistance and blast crisis, and quiescent leukemia stem cells, which are resistant to killing by TKIs^11–13^. Thus, it is necessary to identify additional therapeutic targets to overcome TKI resistance and relapse in CML patients.

The *AF1q/MLLT11* gene was initially identified from acute myeloid leukemia (AML) patients with t(1;11)(q21;q23) chromosomal abnormality^14^. In normal hematopoietic tissues, AF1q expression is largely restricted to T-cell differentiation, but not to mature B and T cells^14^. AF1q is reported to cooperate with the Notch signaling pathway to foster the emergence of bone marrow prothymocytes and to drive subsequent intrathymic maturation toward the T cell lineage^15^. Elevated AF1q expression is found in acute myeloid and lymphoid leukemias and is a poor prognostic biomarker for pediatric AML, adult AML with normal cytogenetics, and adult myelodysplastic syndrome^16–18^. Accumulating evidence shows that AF1q plays a potential proto-oncogenic role in several solid tumors^19–23^. However, the function of AF1q in CML remains unclear.

In the present study, we show that knockdown of AF1q by small interfering RNA (siRNA) suppresses cell survival and sensitizes CML cells or CD34^+^ CML progenitors to IM, whereas elevated AF1q expression contributes to cell growth and protection of CML cells from IM-induced apoptosis. In addition, we confirm that CD44, which is crucial for leukemia stem cell homing, survival, and proliferation^24,25^, is regulated by AF1q. More importantly, inhibition of CD44 activity largely attenuates AF1q-mediated IM resistance in CML.

## Results

### *AF1q* expression is upregulated in CML patients, especially in CD34^+^ CML cells

**Fig. 1:**
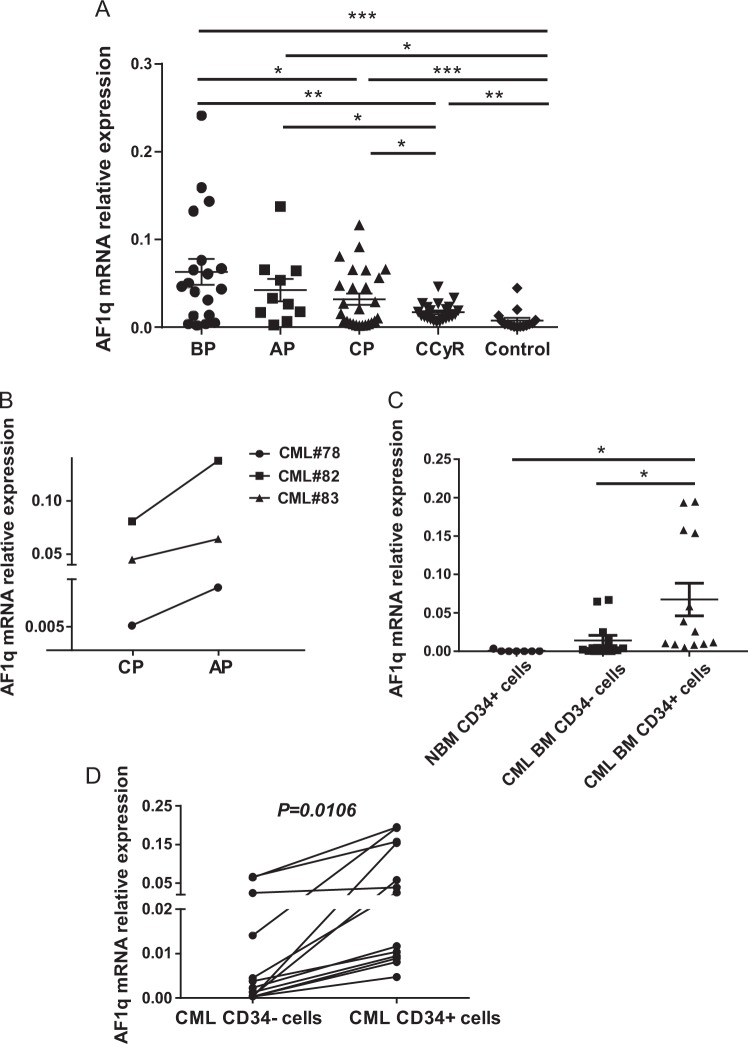
*AF1q* expression was increased in CML patients and CD34^+^ CML cells. **a** *AF1q* expression was measured by qRT-PCR in BMMCs from 77 CML patients (BP, *n* = 19; AP, *n* = 10; CP, *n* = 26; CCyR, *n* = 22) and 15 controls. **b** *AF1q* expression was measured in matched-pair samples acquired from three available follow-up CML patients at the time when they were in CP and when they progressed into AP. **c** *AF1q* levels were evaluated in normal bone marrow CD34^+^ cells from controls (*n* = 7), CML bone marrow CD34^−^ and CD34^+^ cells from newly diagnosed CP CML patients (*n* = 13) by qRT-PCR. β-actin was used as an internal control. **d** *AF1q* levels were analyzed by a paired Student *t* test. *P < 0.05, **P < 0.01, ***P < 0.001

We analyzed *AF1q* expression in bone marrow samples from 77 CML patients (BP, *n* = 19; AP, *n* = 10; CP, *n* = 26; CCyR, *n* = 22) and 15 controls using qRT-PCR (Fig. 1a). Clinical characteristics are summarized in Table S3. *AF1q* mRNA levels were markedly upregulated at all phases of CML compared to controls (*P* < 0.05, Fig. 1a).

Of interest, *AF1q* level seemed to be associated with disease progression. As CML disease progressed into advanced phases, the *AF1q* level increased further. In 5 of 29 (17.24%) samples from BP and AP patients, which were resistant to IM, *AF1q* levels were found to be elevated more than tenfold the average of controls, while only 1 of 26 (3.85%) samples from newly diagnosed CP patients were this elevated (*P* < 0.01, Fig. 1a). *AF1q* expression was higher in patients with AP or BP than in patients with CP, and patients with BP exhibited the highest *AF1q* level (BP and AP vs CP, *P* < 0.05; Fig. 1a). In matched-pair bone marrow samples acquired from three available follow-up CML patients, *AF1q* expression was increased when patients progressed into AP compared to when they were in CP (Fig. 1b). Moreover, *AF1q* expression decreased when CML patients achieved CCyR after successful treatment with IM (CP, AP or BP vs CCyR, *P* < 0.05, Fig. 1a).

Since leukemia stem and progenitor cells are resistant to TKI and are a potential source of disease persistence and relapse, we asked whether AF1q was preferentially expressed in CML progenitor cells. We measured *AF1q* expression in normal bone marrow CD34^+^ cells from seven healthy donors, CML bone marrow CD34^−^ and CD34^+^ cells from 13 newly diagnosed CP CML patients. *AF1q* expression was significantly elevated in CML CD34^+^ cells compared to normal CD34^+^ cells and CML CD34^−^ cells (Fig. 1c, d).

### AF1q knockdown enhances IM sensitivity and promotes IM-induced apoptosis in CML primary and CD34^+^ cells

**Fig. 2:**
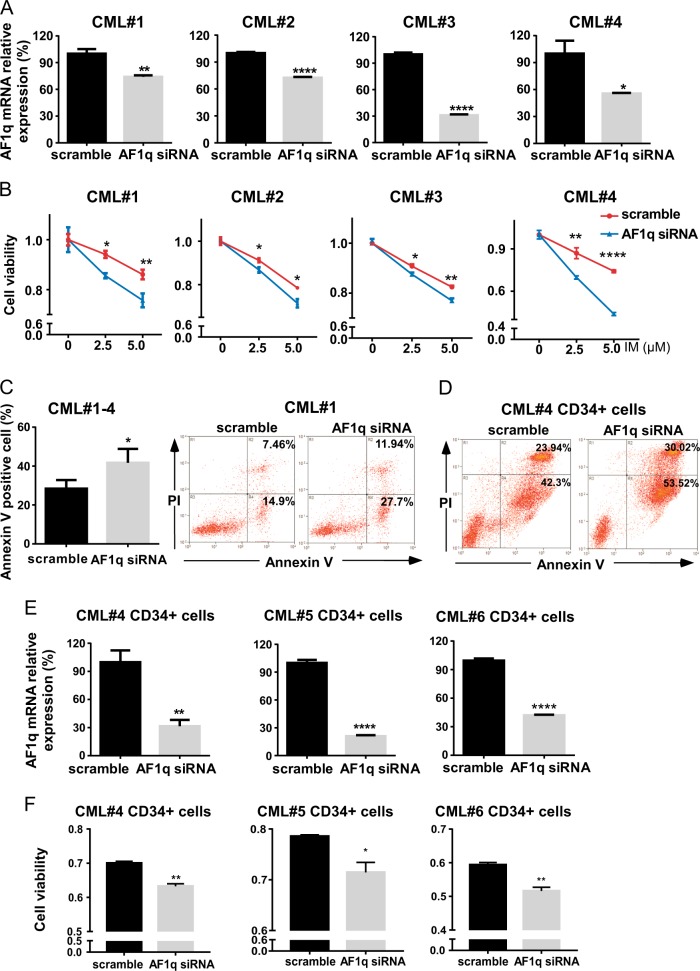
AF1q inhibition enhanced IM sensitivity and promoted IM-induced apoptosis in CML primary and CD34^+^ cells. **a** Primary BMMCs from newly diagnosed CP CML patients (*n* = 4) were transduced with AF1q siRNA or scrambled control sequences. AF1q expression in primary CML cells was estimated by qRT-PCR with β-actin as an internal control. **b** Cell viabilities of primary CML cells transduced with AF1q siRNA or scrambled control were assessed by CCK-8 after IM treatment (48 h, 2.5 or 5 μM). **c** Primary CML cells transduced with AF1q siRNA or scrambled control were cultured with IM (5 μM, 48 h) and apoptosis was measured by flow cytometry with dual staining of Annexin V and PI. Representative dot plots and graphs are shown. **d** CD34^+^ CML cells from CP CML patients transduced with AF1q siRNA or scrambled control were treated with IM (10 μM, 48 h) and apoptosis was measured by flow cytometry with dual staining of Annexin V and PI. Representative dot plots of apoptosis are shown. **e** CD34^+^ cells isolated from newly diagnosed CP CML patients (*n* = 3) were transduced with AF1q siRNA or scrambled control sequences and *AF1q* expression was estimated by qRT-PCR and normalized to β-actin. **f** Cell viabilities of CD34^+^ CML cells transduced with AF1q siRNA or scrambled control were assessed by CCK-8 after IM treatment (48 h, CML#4 5 μM, CML#5 and #6 10 μM). Mean ± SEM. Student *t* test. *P < 0.05, **P < 0.01, ***P < 0.001, ****P < 0.0001

To look for the underlying effects of AF1q in CML, we transduced primary bone marrow cells from four untreated CP CML patients with AF1q specific siRNA and scrambled control. Inhibition was verified by qRT-PCR, which showed that the AF1q expression was significantly suppressed by AF1q siRNA (Fig. 2a). Downregulation of AF1q sensitized primary CML cells to IM. When primary CML cells were treated with IM for 48 h, cell viability decreased at a greater rate in cells transduced with AF1q siRNA (Fig. 2b). Similarly, AF1q suppression enhanced IM-induced cell apoptosis in primary CML cells (Fig. 2c).

As stated, *AF1q* was highly expressed in CD34^+^ CML cells. Based on our findings above, we hypothesized that AF1q might also enhance survival of CD34^+^ CML cells. To test this, we first isolated CD34^+^ cells from three newly diagnosed CP CML patients and transduced these cells with AF1q siRNA or scrambled control. *AF1q* expression in CD34^+^ CML cells was significantly reduced by AF1q siRNA (Fig. 2e). Second, we investigated the role of AF1q inhibition in growth and apoptosis of CD34^+^ CML cells. After treatment with IM for 48 h, cell viability dropped faster in CD34^+^ CML cells transduced with AF1q siRNA compared with scrambled control (Fig. 2f). Apoptosis of CD34^+^ CML cells induced by 10 μM of IM was also enhanced by AF1q inhibition (Fig. 2d). These results indicate that downregulation of AF1q can make CD34^+^ CML cells more sensitive to IM treatment.

Taken together, our results suggest that AF1q knockdown suppresses cell survival and enhances IM sensitivity in both CML primary and CD34^+^ cells.

### AF1q overexpression promotes proliferation and protects CML cells from IM-induced apoptosis in vitro

**Fig. 3:**
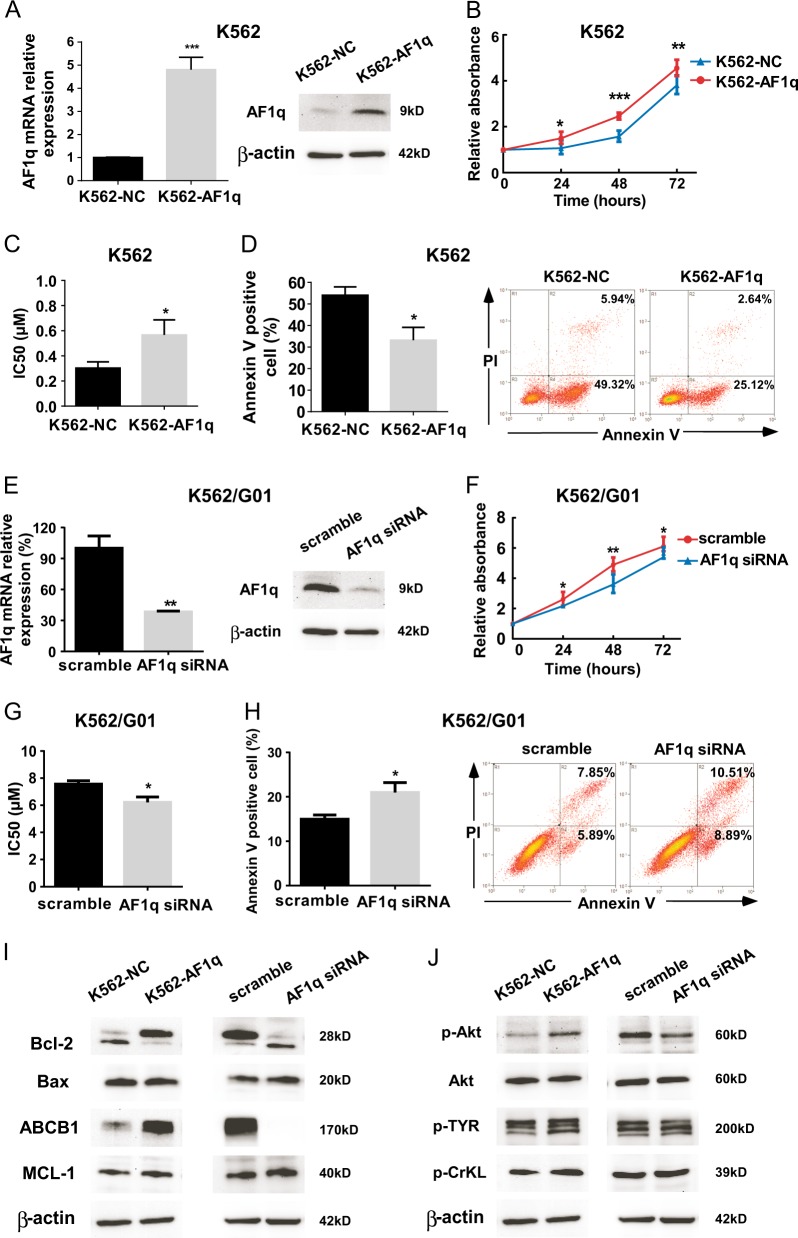
AF1q overexpression promoted cell survival in the IM-sensitive CML cell line K562 and AF1q inhibition impaired cell survival in the IM-resistant CML cell line K562/G01. **a** K562 cells were infected with lentiviruses harboring AF1q (K562-AF1q) or negative control (K562-NC) and AF1q expression was determined by qRT-PCR and western blot. AF1q levels were normalized to the expression of β-actin. **b** Proliferation of K562-AF1q and K562-NC cells were assessed by CCK-8 assays, and proliferation rates at 24, 48, and 72 h were calculated compared to the absorbance at 0 h. **c** IC_50_ values of K562-AF1q and K562-NC cells were calculated according to the cell growth inhibition after 48 h treatment with serial dilutions of IM (0, 0.1, 0.2, 0.4, 0.6, 0.8 μM). **d** K562-AF1q and K562-NC cells were treated with IM (0.4 μM, 48 h), and the percentage of apoptotic cells was measured by Annexin V and PI staining using flow cytometry. Representative dot plots and graphs are shown. **e** K562/G01 cells were transduced with AF1q siRNA or scrambled control sequences and AF1q expression was determined by qRT-PCR and western blot. AF1q levels were normalized to β-actin. **f** Proliferation of K562/G01 cells transduced with AF1q siRNA or scrambled control was assessed by CCK-8 assays, and proliferation rates at 24, 48, and 72 h were calculated compared to the absorbance at 0 h. **g** IC_50_ values for K562/G01 cells transduced with AF1q siRNA or scrambled control were calculated according to the cell growth inhibition after 48 h treatment with serial dilutions of IM (0, 2.5, 5.0, 7.5, 10.0, 12.5 μM). **h** K562/G01 cells transduced with AF1q siRNA or scrambled control were cultured with IM (5 μM, 48 h) and apoptosis was measured by flow cytometry with dual staining of Annexin V and PI. Representative dot plots and graphs are shown. Mean ± SEM. Student *t* test. **i** Expression of several proteins related to apoptosis and drug resistance were measured by western blot. **j** Activity of the PI3K/AKT pathway and phosphorylation levels of p-TYR and p-CrKL were was measured by western blot. *P < 0.05, **P < 0.01, ***P < 0.001

To further study whether altered biological consequences of CML cells were caused by AF1q expression, we used a pLOC-AF1q lentivirus vector to overexpress AF1q in an IM-sensitive CML cell line, K562. Infection with AF1q-expressing lentiviruses increased AF1q expression in K562 cells (Fig. 3a, S5).

We then investigated the effects of AF1q overexpression on cell proliferation and IM treatment. Overexpression of AF1q increased proliferation of K562 cells (Fig. 3b), and the IC_50_ value of IM for K562-AF1q cells was significantly higher than controls (Fig. 3c). Correspondingly, AF1q overexpression reduced cell apoptosis induced by IM (Fig. 3d). These results suggest that AF1q can promote proliferation and protect CML cells from IM-induced apoptosis.

Accordingly, we used K562/G01, a K562-homologous but IM-resistant cell line to validate the effects of AF1q inhibition. The inhibitory effect of AF1q siRNA was verified by qRT-PCR and western blot analysis (Fig. 3e, S5). Proliferation of K562/G01 cells was significantly decreased as a result of AF1q suppression (Fig. 3f). Meanwhile, the inhibition of AF1q enhanced the sensitivity of K562/G01 cells to IM, with a modest but significant decrease in the IC_50_ value from 7.561 ± 0.248 to 6.220 ± 0.229 μM (*P* < 0.05, Fig. 3g). Similarly, AF1q suppression enhanced IM-induced cell apoptosis in K562/G01 cells (Fig. 3h).

We also explored the relationship between *AF1q* expression and *BCR/ABL* transcripts in 72 newly diagnosed CP CML patients (patient characteristics summarized in Table S4) and no significant correlation was found between them (*P* *=* 0.3789). Moreover, when we examined CrKL and TYR, two of BCR-ABL downstream phosphorylation targets, their phosphorylation levels did not change significantly in K562 cells with increased or decreased AF1q expression (Fig. 3j, S5). These findings suggest a kinase-independent mechanism of AF1q in CML.

Furthermore, we measured the expression of several proteins related to apoptosis and drug resistance by western blot. Results showed elevated ABCB1 as well as a higher ratio of Bcl-2/Bax in K562-AF1q cells (Fig. 3i, S5), which might explain the attenuated apoptosis and drug sensitivity after AF1q overexpression. Besides, an enhanced expression of phosphorylated Akt (p-Akt) was found in AF1q-overexpressed K562 cells by western blot (Fig. 3j, S5), which could match one of the results from the gene-wide microarray assay.

### AF1q overexpression enhances engraftment of CML cells in vivo

We next evaluated the effects of AF1q on cell repopulating capacity in vivo. K562 cells infected with lentiviruses harboring AF1q or negative control were transplanted into sublethally irradiated NOD/SCID mice by tail vein injection. K562 cell engraftment into bone marrow and spleens was assessed 4 or 8 weeks after injection by examining human CD33 expression (a surface marker of K562). Mice inoculated with AF1q-overexpressing K562 cells had more CD33^+^ cells in the spleen than those inoculated with control K562 cells after both 4 and 8 weeks, indicating enhanced short-term and long-term engraftment, respectively (Figure S2A-B). Meanwhile, CD33^+^ cells were elevated in bone marrow only after 8 weeks (Figure S2A-B). Furthermore, K562 cell engraftment was higher in spleen than in bone marrow after transplantation.

Additionally, we used 12 mice for each group to measure survival. Two mice died in the control group while seven mice died in the AF1q-overexpressing group after 150 days of observation (Figure S2C, *P* = 0.0576). These results reveal that elevated AF1q expression enhances engraftment of CML cells in vivo.

### AF1q-overexpressing CML cells have a distinct gene expression signature

**Fig. 4:**
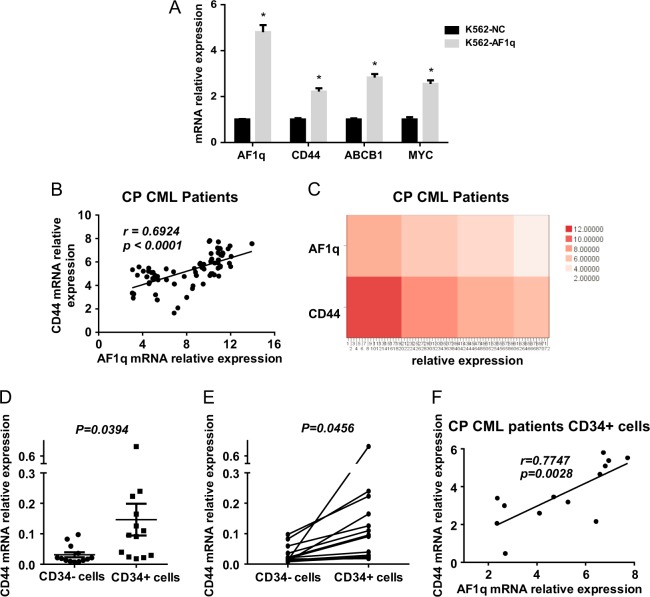
Correlation between AF1q and CD44 expression in CML cells. **a** Three differentially expressed genes (*CD44, ABCB1*, and *MYC*) and AF1q were detected by qRT-PCR to verify microarray analyses. **b** *AF1q* and *CD44* levels in bone marrow samples from newly diagnosed CP CML patients (*n* = 72) were determined by qRT-PCR and normalized to β-actin. A positive correlation was observed between *AF1q* and *CD44* expression in CP CML patients. **c** Heat map for *AF1q* and *CD44* mRNA expression in CP CML patients (*n* = 72). Columns represented CML patients and rows represented *AF1q* and *CD44* expression. Colors from dark to light indicated high to low relative expression of *AF1q* and *CD44*. **d, e** *CD44* levels were evaluated in CD34^+^ and CD34^−^ cells isolated from newly diagnosed CP CML patients (*n* = 13) by qRT-PCR and normalized to β-actin. Results were analyzed by unpaired (**d**) and paired (**e**) Student *t* tests. **f** A positive correlation was observed between *AF1q* and *CD44* expression in CD34^+^ CML cells (*n* = 13)

We performed genome-wide microarray assays (Gene Expression Omnibus, accession: GSE84842) in AF1q-overexpressing K562 cells and negative control cells to profile differentially expressed genes that might be involved in AF1q regulation. We found that 582 genes were differentially expressed after AF1q overexpression, including 465 upregulated and 117 downregulated genes (Figure S3), which formed a distinct signature. Next we examined the expression of multiple AF1q downstream targets from the microarray (*CD44, ABCB1*, and *MYC*) by qRT-PCR in K562 cells. These leukemia-related genes were also found elevated in K562-AF1q cells (Fig. 4a). Among them, CD44 stood out, since it has been found elevated in several kinds of leukemias, including CML^26^, and plays crucial roles in leukemia stem cell maintenance and self-renewal^25,27–29^. Taking all of our results and the previous reports into consideration, we speculated that CD44 is a downstream effector of AF1q in CML.

### CD44 is a potential target of AF1q in CML

To verify this speculation, we first examined *CD44* and *AF1q* expression in bone marrow samples from 72 newly diagnosed CP CML patients by qRT-PCR. A significant positive correlation was observed between *AF1q* and *CD44* expression in these patients (Fig. 4b, c). What is more, we found that CD44 was also significantly elevated in CML CD34^+^ cells compared to CD34^−^ cells (Fig. 4d, e). The qRT-PCR results showed a significant correlation between those two genes within a cohort of 13 CP CML patients’ CD34^+^ cell samples (Fig. 4f). This correlation suggests that AF1q may regulate CD44 expression in CD34^+^ CML progenitor cells.

**Fig. 5:**
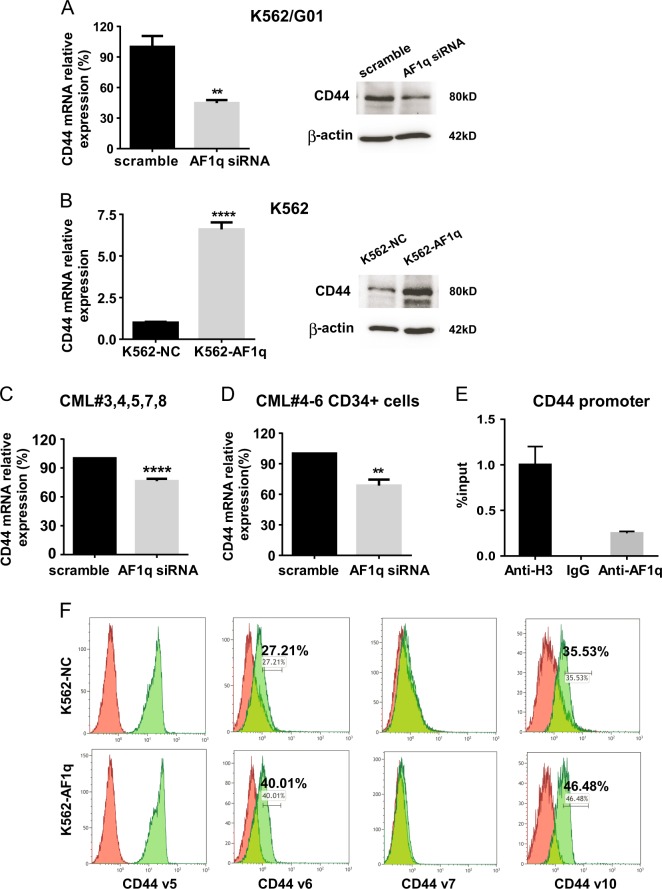
AF1q regulated CD44 expression in CML. **a** CD44 expression in K562/G01 cells transduced with AF1q siRNA or scrambled control was measured by qRT-PCR and western blot. **b** CD44 expression in K562 cells infected with lentiviruses harboring AF1q (K562-AF1q) or negative control (K562-NC) was measured by qRT-PCR and western blot. **c** *CD44* expression was measured by qRT-PCR in primary BMMCs from CP CML patients (*n* = 5) transduced with AF1q siRNA or scrambled control. **d** *CD44* expression in CD34^+^ CML cells (*n* = 3) transduced with AF1q siRNA or scrambled control was measured by qRT-PCR. **e** ChIP analysis of AF1q binding to CD44 promoter region. **f** Frequencies of CD44 v5, v6, v7, and v10 were measured by flow cytometry in K562 cells infected with AF1q lentiviruses. Red represents K562-NC and green represents K562-AF1q. **P < 0.01, ****P < 0.0001

To further confirm that AF1q modulates CD44 expression, we analyzed CD44 expression in CML cells transduced with AF1q siRNA or AF1q-expressing lentiviruses. AF1q inhibition in primary CML cells and K562/G01 cells reduced CD44 expression (Fig. 5a, c and S5). Conversely, AF1q overexpression in K562 cells triggered CD44 activation (Fig. 5b, S5). Furthermore, we transfected CD34^+^ CML cells with AF1q siRNA. As expected, AF1q downregulation suppressed *CD44* expression (Fig. 5d). This is the first experimental evidence that CD44 is positively regulated by AF1q in primary and CD34^+^ CML cells.

A chromatin immunoprecipitation (ChIP) assay was performed to check out the possible direct transcriptional regulation of CD44 by AF1q. As shown in Fig. 5e, AF1q occupied the CD44 promoter region in K562 cells, indicating AF1q is recruited to the CD44 promoter, thereby facilitating the transcription of CD44.

The CD44 isoforms have been categorized as the standard isoform (CD44s) and the variant isoforms (CD44v), both known stem cell markers. CD44s is highly expressed in many cells and most abundantly in cells of the hematopoietic system (100% positive in K562 cells, data not shown), whereas CD44v expression is more restricted^30^. Here, to identify which variants are regulated by AF1q in CML, we tested four variants (CD44 v5, v6, v7, and v10) that had previously been reported to be expressed in various leukemias^31^. Based on our flow results, CD44 v6 and v10 levels were elevated in K562 cells transduced with AF1q-expressing lentiviruses (Fig. 5f). CD44 v5 was constitutively expressed, while CD44 v7 was not expressed in K562 cells transduced with lentiviruses harboring either AF1q or scrambled control (Fig. 5f).

### Inhibition of CD44 activity attenuates AF1q-mediated IM resistance in CML

**Fig. 6:**
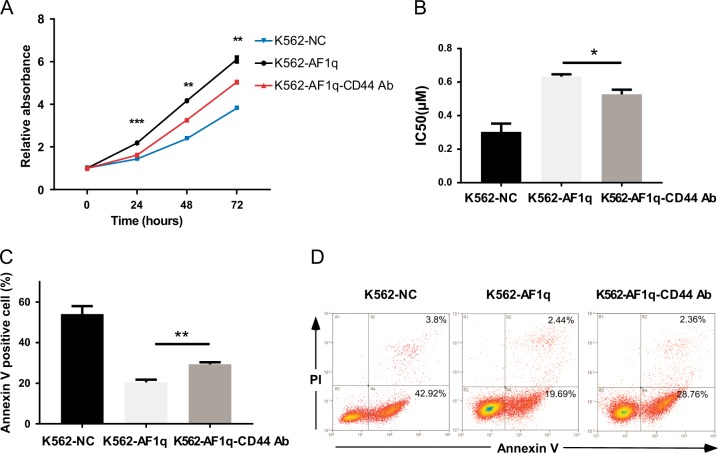
CD44 inhibition attenuated AF1q function in CML. K562 cells infected with AF1q-expressing lentiviruses were incubated with (AF1q-CD44 Ab) or without (AF1q) a CD44 mAb, A3D8. K562 cells infected with negative control lentivirus were used as negative controls (NC). **a** Proliferation was measured by CCK-8 assays and proliferation rates at 24, 48, and 72 h were calculated compared to absorbance at 0 h. **b** IC_50_ values were calculated according to cell growth inhibition after IM treatment (0, 0.1, 0.2, 0.4, 0.6, 0.8 μM; 48 h). **c, d** Apoptotic cells were detected by Annexin V and PI staining using flow cytometry after IM treatment (0.4 μM, 48 h). Representative dot plots and graphs are shown. Mean ± SEM. Student *t* test. *P < 0.05, **P < 0.01, ***P < 0.001

To determine whether AF1q-mediated alterations of CML cells are mainly caused by CD44, we used a specific CD44 monoclonal antibody, A3D8, to block CD44 activity in K562-AF1q cells. CD44 dysfunction resulted in significantly slowed proliferation of K562-AF1q cells, which was accelerated by AF1q overexpression (Fig. 6a). Inhibition of CD44 in IM-treated K562-AF1q cells decreased the IC_50_ value from 0.633 ± 0.013 to 0.527 ± 0.027 μM (*P* < 0.05, Fig. 6b). Similarly, blocking CD44 activity increased IM-induced apoptosis in K562-AF1q cells (Fig. 6c, d). These results reveal the pro-survival activities of CD44 in CML cells. Importantly, we demonstrate that CD44 inhibition partially attenuates AF1q-mediated survival promotion and protection of CML cells from IM treatment.

## Discussion

Development of genetic and molecular characterizations allow us to practice targeted therapy in leukemia, among which the most dramatic example is TKI in CML. Imatinib (IM) is the first successful molecular drug specifically targeting the aberrantly expressed BCR-ABL oncoprotein in CML cells, improving the estimated 8-year survival rate to ~88%^32^. However, not all CML patients benefit greatly or equally from it. TKI resistance mediated by *BCR-ABL* gene amplifications and BCR-ABL kinase domain mutations frequently occurs in CML patients and neither the second nor the third generation of TKIs conquers it completely^33^. Even CML patients with undetectable *BCR-ABL* transcripts are at risk for rapid disease recurrence after discontinuation of TKI therapy^7^. Leukemia stem cells considered responsible for drug resistance and relapse of CML are especially resistant to TKI therapy, despite effective inhibition of BCR-ABL tyrosine kinase activity^13^. Given these findings, investigation of alternative strategies to control CML by BCR-ABL kinase-independent pathways is important.

The *AF1q* gene, originally considered an oncogenic factor, promoted tumor growth and metastasis in breast cancer and was involved in thyroid tumorigenesis^19–23^. Tse et al. reported that 84% of pediatric AML patients had elevated *AF1q* expression and that increasing *AF1q* expression was associated with decreased overall survival^16^. Moreover, miR-29b directly regulated AF1q in AML, and patients with low miR-29b/elevated AF1q expression had poor overall survival^34^.

The present study found that CML patients expressed higher *AF1q* levels than controls and *AF1q* expression increased during CML development. CML patients in AP and BP that were resistant to IM had higher *AF1q* levels than those in CP. *AF1q* expression increased greater than 15-fold in a higher proportion of AP and BP patients than in CP patients. Using follow-up AP/CP samples from the same patients, we demonstrated that *AF1q* expression was increased when patients progressed into AP compared to CP. These data suggest that AF1q may be a new indicator of disease progression and may play an important role in TKI resistance.

We then explored the biological effects of AF1q in CML by gain- and loss-of-function experiments. In this work, we demonstrated that upregulation of AF1q expression promoted proliferation, decreased the sensitivity of CML cells to IM, and protected cells from IM-induced apoptosis. Conversely, AF1q downregulation enhanced IM sensitivity, induced apoptosis, and suppressed growth of CML cells. These results were consistent with previous reports describing that enhanced AF1q expression promoted cell proliferation, migration, and chemo-resistance in breast cancer^19–21^. However, other reports demonstrated overexpression of AF1q increased apoptosis in ovarian cancer and squamous carcinoma cell lines^35–37^. The reason for this discrepancy is unclear, but may be due to the differences in tumor models. In addition, we found that AF1q overexpression increased engraftment of CML cells in vivo and reduced survival of mice transplanted with CML cells, although this difference did not reach statistical significance (*P* = 0.0576). Taken together, these findings reveal novel roles of AF1q in modulating CML cell survival and response to IM. Thus, AF1q represents a potential therapeutic target for CML.

CML leukemia stem cells are insensitive to kinase inhibitors and responsible for CML relapse. TKI therapies target proliferating mature cells only, therefore leaving the reversibly quiescent leukemia stem cell pool to repopulate the original disease^38,39^. Further, these leukemia stem cells are independent of BCR-ABL kinase activity for survival^40^. Additional therapy may be required to target leukemia stem cells for disease eradication. To determine whether *AF1q* is a potential survival-related gene for CML leukemia stem cells, we measured its expression in CD34^+^ progenitor cells from newly diagnosed CP CML patients. We found that *AF1q* was significantly elevated in CD34^+^ CML cells compared with CD34^−^ CML cells and normal CD34^+^ cells. More importantly, AF1q knockdown in CD34^+^ CML cells increased sensitivity and apoptosis in response to IM treatment. Consistent with the CML cell results, we preliminarily verified our hypothesis that AF1q is a potential therapeutic target for CML leukemia stem cells with limited experiments. This hypothesis should be further explored using a larger patient cohort and more biological experiments.

The abundant microarray data offered us the opportunity to explore important biological processes regulated by AF1q in CML. A gene ontology analysis was performed, which revealed roles of AF1q in transcription regulation, signal transduction, adhesion, apoptosis, and more (Figure S4A). Functional annotation analysis was also performed to generate a GeneSignalNetwork, such as “activation”, “binding/association”, and “inhibition’, which clearly showed relationships among differentially expressed genes (Figure S4B). The gene pathway analysis based on the KEGG database better classified the differentially expressed genes and represented the enrichment results as a PathwayRelationNetwork (Figure S4C), among which CML and AML pathways were identified as activated.

We focused on CD44 as the downstream target for two reasons. First, because microarray analysis showed elevated *CD44* expression following AF1q upregulation and, second, because CD44 was demonstrated to be crucial for leukemia stem cell maintenance and self-renewal. Our results revealed a positive correlation between *AF1q* and *CD44* expression in a cohort of 72 newly diagnosed CP CML patients. Upregulated or downregulated AF1q caused corresponding changes in CD44 expression. These results were consistent with a recent study in which AF1q activated CD44 in breast cancer^20^. Moreover, we blocked the function of CD44 in AF1q-overexpressing CML cells and found attenuated survival activities and sensitivity to IM in these cells, which partially counteracted the deleterious effects of AF1q. These data indicate that the effects of AF1q on CML cell survival and IM resistance are mediated, at least in part, via regulation of CD44 expression.

Interestingly, CD44 upregulation has been linked to enhanced metastatic potential and poor prognosis in several types of cancer^41,42^. As it relates to leukemia, CD44 expression promoted both CML and AML stem cell maintenance in mouse models^25,27,28^. Recently, Holm et al. demonstrated that CD44 was upregulated during CML blast crisis transformation and CD44 inhibition reduced blast crisis leukemia stem cell self-renewal^29^. In the current study, *CD44* expression level was also found upregulated and positively related to *AF1q* expression in CD34^+^ CML progenitors. *AF1q* knockdown in CD34^+^ CML progenitors reduced *CD44* expression. Taken together, these data suggest that AF1q regulates CD44 expression in CML progenitors, showing a potential mechanism for leukemia stem cell maintenance.

The present study has shown that *AF1q* expression is elevated in CML patients and its expression positively correlates with CML disease progression. AF1q supports cell survival, decreases IM sensitivity, and reduces apoptosis of CML cells by modulating CD44. Inhibition of AF1q sensitizes CD34^+^ CML cells to TKI treatment. Thus, targeting AF1q or CD44 may offer a promising therapeutic strategy for this disease.

## Materials and methods

### Patient samples and CD34^+^ cell isolation

Bone marrow samples from 149 CML patients and 15 control donors were obtained following informed consent at Qilu Hospital, Shandong University. CML patient samples were collected at different time points of the disease, including CP without prior IM treatment, accelerated phase (AP), blastic phase (BP), and CCyR after successful IM treatment. Control samples were obtained from donors who were free from any clonal bone marrow disorder. Mononuclear cells from bone marrow aspirates were separated by Ficoll-Paque Plus (Pharmacia LKB Biotechnology) density gradient centrifugation. Normal bone marrow CD34^+^ cells and CML bone marrow CD34^+^ cells were isolated by using a human CD34 Microbead Kit (Miltenyi Biotec) and the purity of CD34^+^ cells was above 90% as assessed by flow cytometry (Figure S1).

### Cell lines and cell culture

The human CML cell line K562 and IM-resistant cell line K562/G01 were purchased from the Institute of Hematology & Blood Diseases Hospital, Chinese Academy of Medical Sciences & Peking Union Medical College, Tianjin, China. Cells were cultured in RPMI 1640 medium supplemented with 10% fetal bovine serum (Gibco) and 1% penicillin–streptomycin (Invitrogen) in a humidified incubator at 37 °C with 5% CO_2_. For K562/G01, IM was added to the complete culture medium with final concentration of 4 μM until 2 weeks before the assay.

### siRNAs, lentiviruses, and gene transfer

The AF1q-specific siRNAs and scrambled control sequences were synthesized by RiboBio (Guangzhou, China; Table S1) and transfected into CML cells using Lipofectamine 2000 reagent (Invitrogen). The AF1q-expressing lentivirus vector pLOC-AF1q and its negative control pLOC-NC were generously provided by Dr. William Tse (University of Louisville School of Medicine, Louisville, KY). Methodology of the lentivirus production are detailed in the supplemental experimental procedures. K562 cells infected with lentiviruses harboring AF1q (K562-AF1q) or negative control (K562-NC) were selected by 10 µg/mL blasticidin (Selleck).

### Engraftment of CML cells in immunodeficient mice

K562 cells infected with lentiviruses harboring AF1q or negative control (5 × 10^6^ cells/mouse) were injected via tail vein into irradiated (1.5 Gy) NOD/SCID immunodeficient mice (Beijing HFK Bioscience Co., Ltd., Beijing, China). Mice were euthanized after 4 or 8 weeks. Bone marrow contents of femurs and spleen cells were checked for human K562 cell engraftment by staining with anti-human CD33 antibody (BD Biosciences) using flow cytometry. Mice were inspected daily for 150 days and survival was represented using a Kaplan–Meier survival plot.

### CD44 variants analysis and inhibition of CD44 activity

Expression of CD44 variants was analyzed by flow cytometry. Antibodies for CD44 v5, v6, v7, and v10 were purchased from eBioscience. To block CD44 activity in K562-AF1q cells, a specific CD44 monoclonal antibody A3D8 (Sigma-Aldrich) was added to the culture medium with final concentration of 2.5 μg/mL for 48 h.

### Microarray analysis

RNA was extracted from K562 cells infected with lentiviruses harboring AF1q or negative control. Gene expression profiles were detected with the GeneChip Human Gene 1.0 ST Arrays (Affymetrix). Microarray data analysis and gene set enrichment analysis were performed by Gminix Information Technology Co., Ltd. (Shanghai, China).

### ChIP

The SimpleChIP^®^ Enzymatic Chromatin IP Kit (Cell Signaling, Danvers, MA, USA) was used to perform ChIP assays according to the manufacturer’s standard protocol. Chromatin fragments derived from K562 cells were immunoprecipitated with 5 µg AF1q antibody.

### qRT-PCR, western blot, cell assays

Detailed methods for the quantitative real-time polymerase chain reaction (qRT-PCR), western blotting, and cell assays, including cell proliferation, viability and apoptosis, are shown in the supplemental experimental procedures with primer sequences in Table S2.

### Ethic approval and consent to participate

All the procedures involved with patients were approved by the Medical Ethics Committee of Qilu Hospital according to institutional guidelines and Declaration of Helsinki principles, and patients’ samples were collected with informed consent from participants prior to inclusion in the study. All animal studies were conducted in accordance with the Guide for the Care and Use of Laboratory Animals published by the US National Institutes of Health and Shandong University.

### Statistics

Data are presented as mean ± standard error of the mean (SEM). Significant differences were determined using the Student *t* test, *χ*^2^ test, and Spearman correlation test.

## Electronic supplementary material

supplemental material
