# Supplementary material for: Novel AF1q/MLLT11 favorably affects imatinib resistance and cell survival in chronic myeloid leukemia
Source: Cell Death Dis. 2018 Aug 28;9(9):855. doi: 10.1038/s41419-018-0900-7 (PMC6113287; doi:10.1038/s41419-018-0900-7)
Supplement: Supplementary file 1 — supplemental material [file 41419_2018_900_MOESM1_ESM.pdf]

Supplemental Information

Supplemental figures and legends

Figure S1

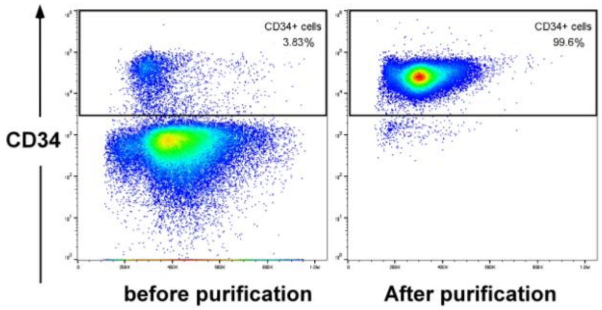

Figure S1. CD34$^+$ cell purity. CD34$^+$ cells were isolated from BMMCs of healthy controls and CML patients using a human CD34 Microbead Kit. CD34$^+$ cell purity was assessed by flow cytometry.

Figure S2

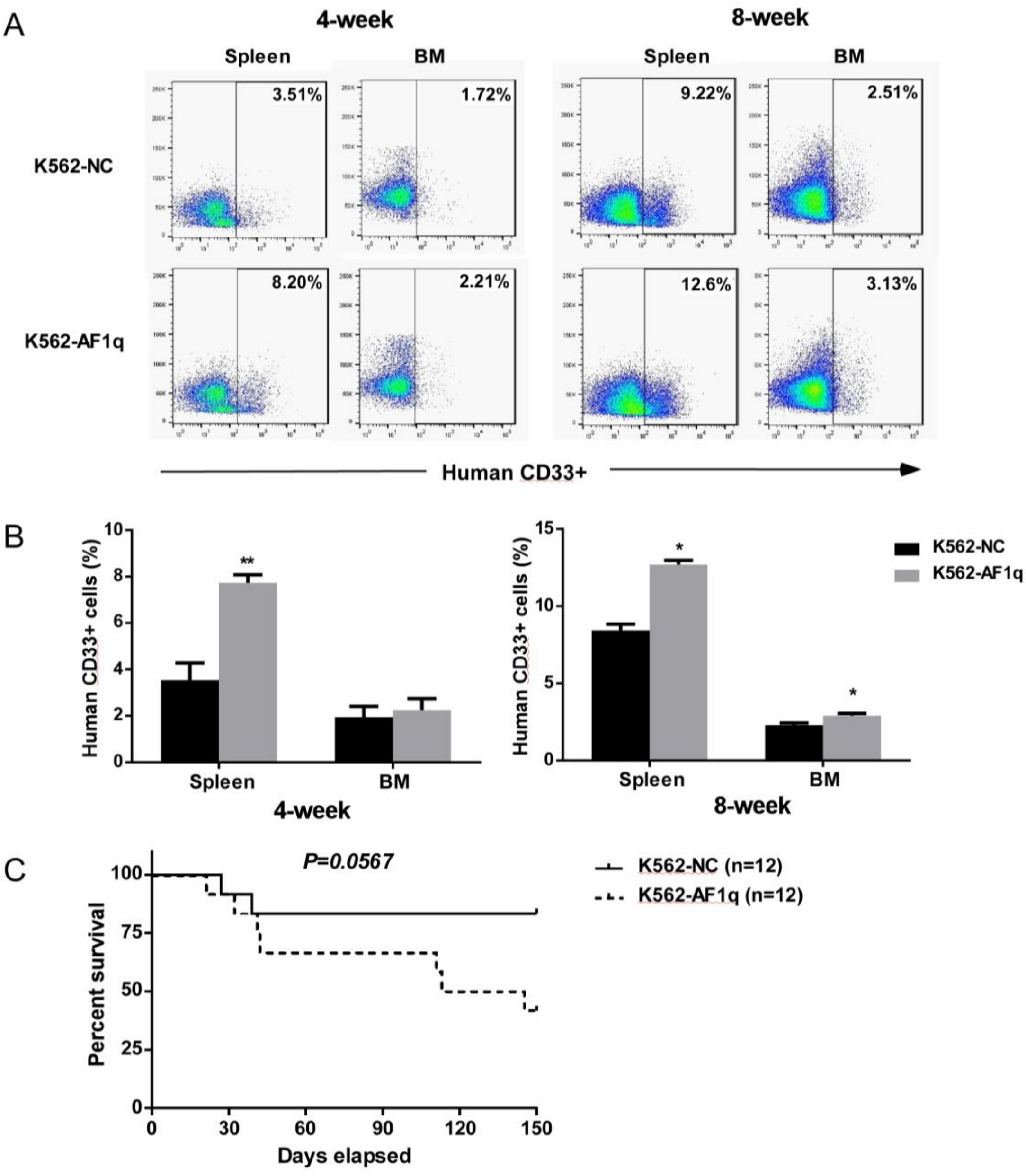

Figure S2. AF1q overexpression increased engraftment of CML cells *in vivo*. (**A-B**) K562-AF1q and K562-NC cells were injected into sublethally irradiated NOD/SCID mice. The percentage of engrafted human $CD33^+$ cells in bone marrow and spleens of recipient mice were detected by flow cytometry 4 or 8 weeks after transplantation. **(C)** Kaplan-Meier survival analysis of NOD/SCID mice transplanted with K562-AF1q and K562-NC cells. $^*P < 0.05$, $^{**}P < 0.01$.

Figure S3

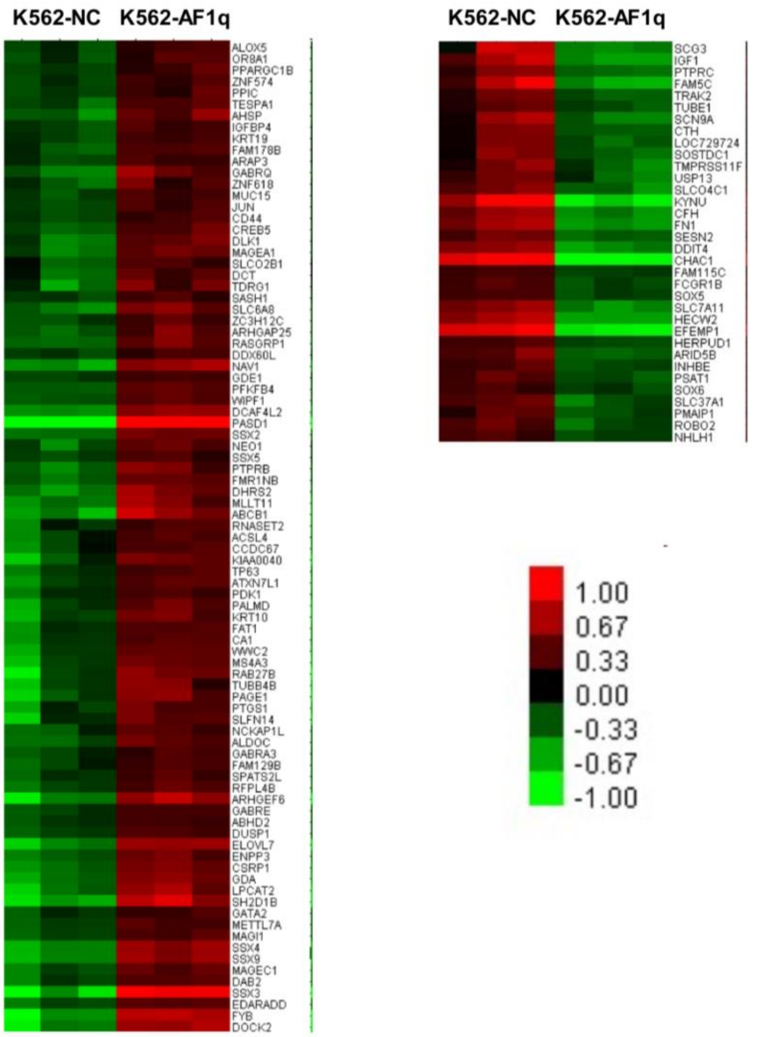

Figure S3. Differentially expressed genes in AF1q-overexpressing CML cells. Heat map for microarray assays in AF1q-overexpressing K562 cells (K562-AF1q) and negative control cells (K562-NC). Colors from red to green indicate high to low relative expression. N = 3.

Figure S4

A

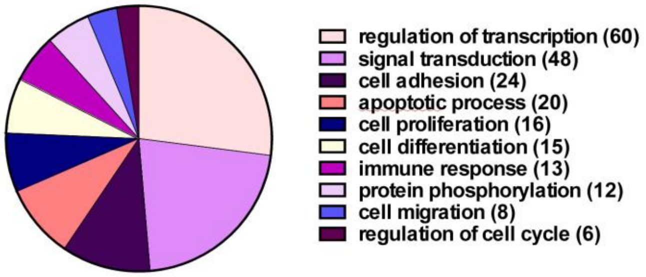

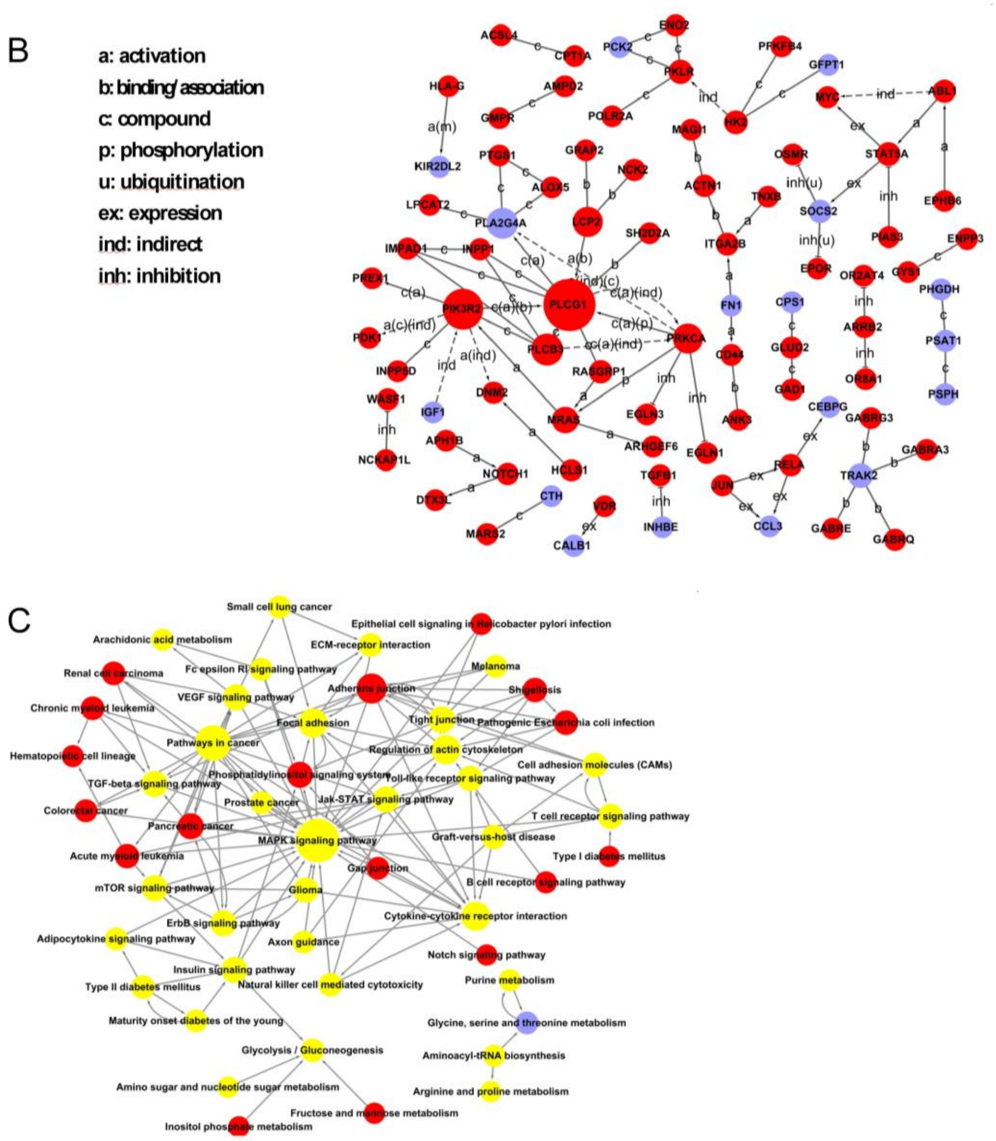

Figure S4. Microarray analysis in AF1q-overexpressing CML cells. Microarray assays in AF1q-overexpressing K562 cells and negative control cells were performed. (**A**) Gene ontology enrichment analysis of differentially expressed genes. The number of genes with a significantly changed expression is shown in parentheses. (**B**) GeneSignalNetwork depicting connections among differentially expressed genes. Red and blue dots represent significantly upregulated and downregulated genes, respectively. The size of dots reflects significance. (**C**) PathwayRelationNetwork depicting connections among differentially expressed KEGG gene sets. Red and blue represent upregulated and downregulated genes, respectively, while yellow includes both. Dot size represents significance.

Figure S5

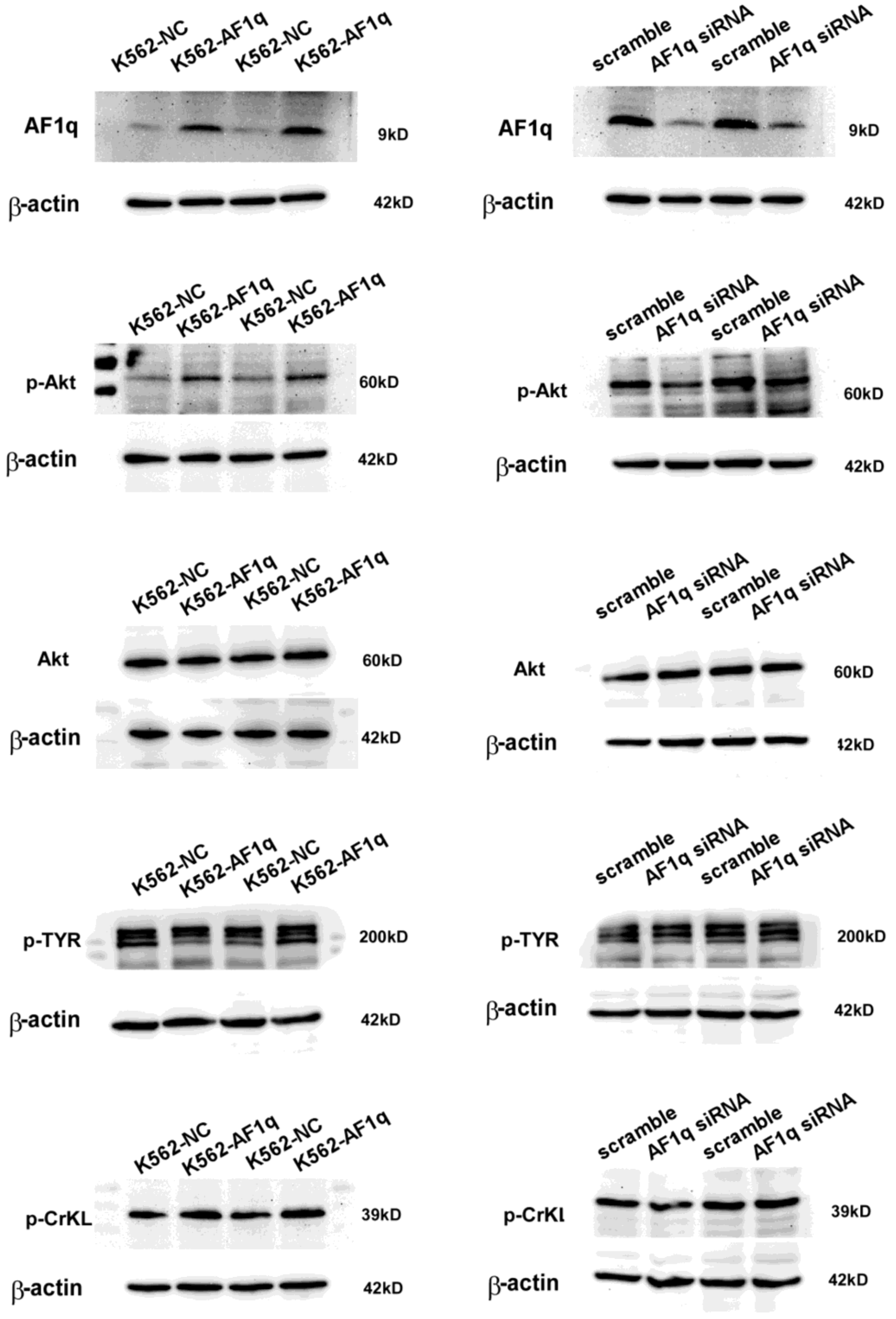

K562-NC
K562-AF1q
K562-NC
K562-AF1q
scramble
AF1q siRNA
scramble
AF1q siRNA
AF1q
9kD
β-actin
42kD
p-Akt
60kD
Akt
p-TYR
200kD
p-CrKL
p-CrKl
39kD

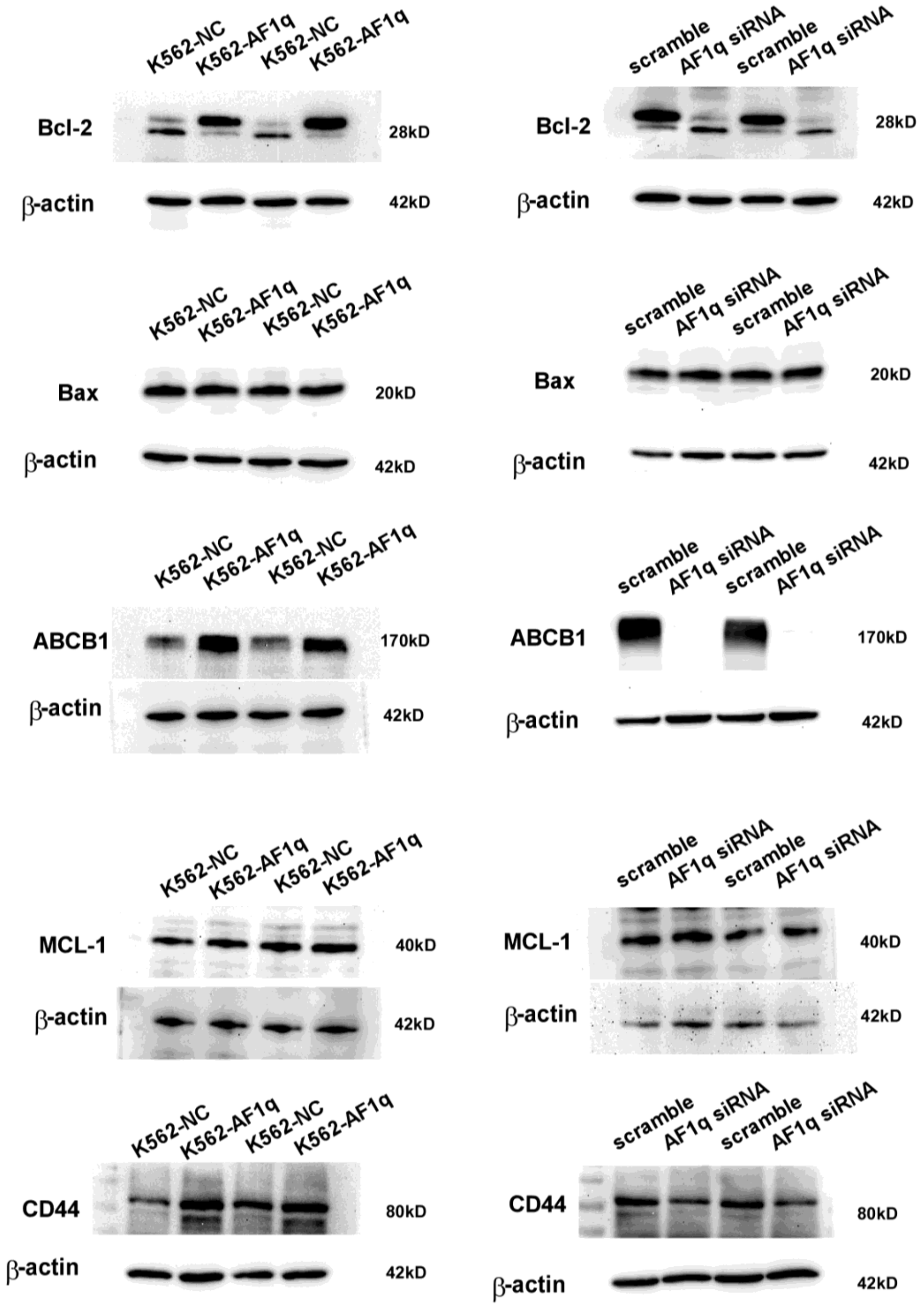

Figure S5. Western blot duplicates related to Figure 3A, E, I, J and Figure 5A, B. Here presented duplicates of all the western blots showed in the manuscript.

Supplemental Tables

Table S1. Specific siRNAs for AF1q knockdown in CML cells

siRNAs	Product number (company), sequence (5' to 3')
AF1q siRNA #1	siG1252890920, CCUGUGAGUAGCCAGUACA dTdT
AF1q siRNA #2	siG1252890937, GGAGGAAAUAUUAUGGAAA dTdT
AF1q siRNA #3	siG1252890946, GCAUUUGCAAUACGUAAUA dTdT
Scrambled control	siN05815122147, siR-RiboTM Negative Control

Table S2. Primers used for qRT-PCR of CML cells

Primer Name	Sequence (5' to 3')
AF1q-Foward	GGACCCTGTGAGTAGCCAGTA
AF1q-Reverse	CTTGCCCGATCATTTTGCCA
CD44-Foward	AACCCTTGCAACATTGCCTGA
CD44-Reverse	GCTTCCAGAGTTACGCCCTTGA
β-actin-Foward	CCTTCCTGGGCATGGAGTCCTG
β-actin-Reverse	GGAGCAATGATCTTGATCTTC

Table S3. Clinical characteristics of CML patients at the time of sampling

Characteristic	BP (n = 19)	AP (n = 10)	CP (n = 26)	CCyR (n = 22)
**Age, y**				
Median	44	50.5	46	33.5
Range	16-73	12-79	25-80	19-53
**Male, n (%)**	14 (74)	9 (90)	18 (69)	17 (77)
**WBC, × 10^9/L**				
Median	58.5	10.4	113.9	4.7
Range	15.3-210.2	8.4-648.6	21.7-466.0	3.3-7.9
**BAS, %**				
Median	1.95	3.70	3.43	0.50
Range	0.1-14.8	0.4-35.1	0.3-10.4	0-1.1
**HGB, g/L**				
Median	85	69.5	109	137
Range	52-130	59-120	86-145	114-160
**PLT, × 10^9/L**				
Median	44.7	193.0	337.0	175.0
Range	10.0-1318.0	23.8-501.0	73.0-2442.0	143.0-213.0
**Blast in peripheral blood, %**				
Median	69	8	1.5	0
Range	15-95	3-17	0-5	0
**Blast in bone marrow, %**				
Median	88	12	3	0
Range	48-98	11-16	1-6	0-1
**Additional chromosome abnormalities, n (%)**				
Yes	3 (16)	3 (30)	0 (0)	0 (0)

AP, accelerated phase; BAS, basophils; BP, blastic phase; CML, chronic myeloid leukemia; CP, chronic phase; CCyR, complete cytogenetic responses; HGB, hemoglobin; PLT, platelets; WBC, white blood cells

Table S4. Clinical characteristics of newly diagnosed CP CML patients

Characteristic	CP CML patients (n = 72)
**Age, y**	
Median	44
Range	16-85
**Male, n (%)**	51 (71)
**WBC, × 10^9/L**	
Median	179.0
Range	10.7-869.5
**BAS, %**	
Median	2.50
Range	0.01-16.10
**HGB, g/L**	
Median	100
Range	71-161
**PLT, × 10^9/L**	
Median	379
Range	142-1474
**Blasts in peripheral blood, %**	
Median	1
Range	0-6
**Blasts in BM, %**	
Median	2
Range	0-8
**BCR-ABL IS, %**	
Median	69.962
Range	17.219-332.773
**Additional chromosome abnormalities, n (%)**	
Yes	2 (2.8)

BAS, basophils; CP, chronic phase; CML, chronic myeloid leukemia; HGB, hemoglobin; IS, international scale; PLT, platelets; WBC, white blood cells

Supplemental Experimental Procedures

Lentivirus production

Lentivirus particles were produced by cotransfecting 293T cells with lentivirus vector pLOC-AF1q or pLOC-NC and pVSV-G, psPAX2 packaging plasmids using Lipofectamine 2000 reagent (Invitrogen) according to the manufacturer's instructions. Supernatants containing lentivirus particles were collected and concentrated by PEG8000 (Sigma-Aldrich).

qRT-PCR

Total RNA was extracted using TRIzol reagent (Invitrogen) and reverse transcription was performed with M-MLV RTase cDNA Synthesis Kit (Takara, Japan). qRT-PCR was conducted on an Applied Biosystems 7900HT System (ABI) with SYBR Green PCR Master Mix (Toyobo, Japan). A comparative CT method was used to analyze gene expression. *β-actin* or *ABL1* were used as internal controls. Primer sequences are available in **Table S2**.

Western blot

CML cells were lysed in protein solubilization buffer and protein extracts were prepared by Total Protein Extraction Kit (BestBio, Shanghai, China) according to the manufacturer's instructions. Proteins were separated by SDS-polyacrylamide gel electrophoresis and transferred to polyvinylidene difluoride (PVDF) membranes. Membranes were blocked with 5% nonfat milk in Tris-buffered saline containing 0.05% Tween-20 (TBST), then labeled with appropriate primary antibodies, followed by horseradish peroxidase-conjugated secondary anti-rabbit antibodies. Protein bands were visualized using FluorChem E Chemiluminescent Western Blot Imaging System (Cell Biosciences). Primary antibodies included anti-AF1q, anti-CD44, and anti-β-actin (Epitomics), as well as anti-Akt, anti-p-Akt, anti-MDR1, anti-MCL1, anti-Bcl-2, and anti-Bax (CST). The horseradish peroxidase-conjugated secondary anti-rabbit antibody was from Santa Cruz Biotechnology. Duplicates of all the western blots in the manuscript are showed in **Figure S5.**

Proliferation, cell viability and apoptosis assays

To assess proliferation, cells were seeded in 96-well culture plates and cultured in an incubator at 37˚C. At each time point of the next 3 days, 10 μL of CCK-8 (BestBio, Shanghai, China) was added to each well, then cells were incubated for 4 h. Absorbance (450 nm) was measured by an automated microplate spectrophotometer (Thermo Scientific).

To measure cell viability, cells were treated with serial dilutions of IM (Santa Cruz Biotechnology) for 48 h. Cell viability was determined by CCK-8 assays and IC_{50} values of IM (the concentration inducing 50% loss of cell viability) were calculated.

To measure apoptosis, cells were cultured in the presence of IM for 48 h, labeled with Annexin V/PI (BestBio, Shanghai, China), and analyzed by a Gallios flow cytometer (Beckman Coulter).
